# Glue Embolization of Gastroesophageal Varices during Transjugular Intrahepatic Portosystemic Shunt (TIPS) Improves Survival Compared to Coil-only Embolization—A Single-Center Retrospective Study

**DOI:** 10.1007/s00270-021-02852-y

**Published:** 2021-05-21

**Authors:** Karsten Wolter, Michael Praktiknjo, Julia Boie, Georges Decker, Jennifer Nadal, Christian Jansen, Wiebke I. Y. Keller, Carsten Meyer, Jonel Trebicka, Ulrike Attenberger, Daniel Thomas

**Affiliations:** 1 Department of Radiology, University Hospital Bonn, Bonn, Germany; 2 Department of Internal Medicine I, University Hospital Bonn, Bonn, Germany; 3 Department of Medical Biometry, Computer Science and Epidemiology, University Hospital Bonn, Bonn, Germany; 4 Department of Marketing / Market Data Analysis, University of Tübingen, Tübingen, Germany; 5 Department of Internal Medicine I, University Hospital Frankfurt, Frankfurt, Germany

**Keywords:** Liver cirrhosis, Portal hypertension, Variceal hemorrhage, Embolization, TIPS

## Abstract

**Purpose:**

To compare the safety and effectiveness of coil versus glue embolization of gastroesophageal varices during transjugular intrahepatic portosystemic shunt (TIPS) creation.

**Materials and Methods:**

In this monocentric retrospective study 104 (males: 67 (64%)) patients receiving TIPS with concomitant embolization of GEV and a minimum follow-up of one year (2008—2017) were included. Primary outcome parameter was overall survival (6 week; 1 year). Six-week overall survival was assessed as a surrogate for treatment failure as proposed by the international Baveno working group. Secondary outcome parameters were development of acute-on-chronic liver failure (ACLF), variceal rebleeding and hepatic encephalopathy (HE). Survival analysis was performed using Kaplan–Meier with log-rank test and adjusted Cox regression analysis.

**Results:**

Indications for TIPS were refractory ascites (n = 33) or variceal bleeding (n = 71). Embolization was performed using glue with or without coils (n = 40) (Group G) or coil-only (n = 64) (Group NG).

Overall survival was significantly better in group G (p = 0.022; HR = -3.333). Six-week survival was significantly lower in group NG (p = 0.014; HR = 6.945).

Rates of development of ACLF were significantly higher in group NG after 6 months (NG = 14; G = 6; p = 0.039; HR = 3.243). Rebleeding rates (NG = 6; G = 3; p = 0.74) and development of HE (NG = 22; G = 15; p = 0.75) did not differ significantly between groups.

**Conclusion:**

Usage of glue in embolization of GEV may improve overall survival, reduce treatment failure and may be preferable over coil embolization alone.

## Introduction

Liver cirrhosis is a major healthcare burden with prevalence values of about 250 per 100.000 inhabitants in western countries. The main etiological factors are alcohol, viral hepatitis and non-alcoholic steatohepatitis (NASH) particularly in western countries [1, 2]. Development of clinical significant portal hypertension (CSPH) in cirrhosis leads to life-threatening complications such as hemorrhage from gastroesophageal varices (GEV) and refractory ascites with consecutive risk of spontaneous bacterial peritonitis, which define acute decompensation episodes and may induce acute-on-chronic liver failure (ACLF) [3–5]. Creation of a transjugular portosystemic stent shunt (TIPS) in variceal hemorrhage and refractory ascites can improve survival in selected patients [6–9]. During TIPS collateral vein embolization may further reduce rebleeding rate [10] and rates of hepatic encephalopathy (HE) [11, 12] development. Moreover, a reduction of the collateral blood flow via varicose vessels may improve TIPS flow and thereby TIPS patency [13, 14]. The hypothesis that embolization of shunts might have an impact on patient outcome, is also supported by the fact that the presence of spontaneous portosystemic shunts (SPSS) has recently been shown to increase rates of decompensation [15]. Recently, a multicenter study showed a significant association of the total area of spontaneous portosystemic shunts (SPSS) with mortality and HE development [16].

Methods of embolization are not standardized, and different materials have been used. A current meta-analysis did not show clear results in favor of one method or the other, underscoring the need for further investigation in this regard [10]. Currently, endovascular coil embolization, glue (n-butyl-2-cyanoacrylate) and vascular plugs are the most common techniques for variceal embolization. The principles of these methods are substantially different. While endovascular coils and plugs obstruct larger afferent vessels, glue penetrates into the peripheral network of shunting vessels and leads to complete occlusion of these collaterals [17].

Vascular plugs and coils seem to be similarly effective [18]; however, a study comparing the effectiveness of glue embolization versus coils has not been performed so far.

The aim of this study was to compare the safety and effectiveness of coil versus glue embolization of gastroesophageal varices during transjugular intrahepatic portosystemic shunt (TIPS) creation.

## Materials and Methods

In this monocentric retrospective study we included 104 patients (Table 1) from the prospective NEPTUN cohort (clinicaltrials.gov identifier NCT03628807) of which several subgroups have previously been reported by Jansen et al. [19], Praktiknjo et al. [20] and Lehmann et al. [21]. These previous reports focused on liver stiffness (collagen type III and IV remodeling) with regard to systemic inflammation markers and liver failure in patients receiving TIPS. None of these reports evaluated different interventional techniques or methods of embolization.

**Table 1:** Patient characteristics (PSPG = portal systemic pressure gradient; TIPS indication A = refractory ascites, B = variceal bleeding; na = not assessed; MOF = multi-organ failure)

	Coil-only (NG)	Glue +—Coil (G)	Sig
No	64	40	
Mean age	58.16 (SD 11.1)	52.43 (SD 13.5)	*P* = 0.02
Male/female	41/23	26/14	*P* = 0.92
MELD	11.65 (SD 5.0)	10.57 (SD 6.5)	*P* = 0.72
*Child–Pugh grading*			
Na	4	5	
A	20	11	
B	25	16	
C	15	8	*P* = 0.71
PSPG pre-TIPS	20.41 (SD 6.93)	20.93 (SD 6.72)	*P* = 0.71
PSPG post-TIPS	8.93 (SD 5.7)	10.23 (SD 5.35)	*P* = 0.25
Bilirubin at TIPS	1.58	1.39	*P* = 0.52
INR at TIPS	1.2	1.2	*P* = 0.74
Creatinine at TIPS	0.95	0.89	*P* = 0.75
TIPS indication (A/B)	A = 23; B = 41	A = 10; B = 30	*P* = 0.25
Hepatic encephalopathy (1 year)	22/64 (34%)	15/40 (38%)	*P* = 0.75
Rebleeding (1 year)	6/64 (9%)	3/40 (8%)	*P* = 0.74
Gastro-/splenorenal shunt	4	3	*P* = 0.55
*GEV grading (endoscopic)*			
Na	14	14	
1	10	5	
2	23	10	
3	14	10	
4	3	1	*P* = 0.81
*Causes of death*			
MOF	3	0	*P* = 0.28
Septic shock	5	2	*P* = 0.70
GEV bleeding	3	2	*P* = 1.0
Liver failure	10	2	*P* = 0.12
Total	21	6	*P* = 0.035

Written patients informed consent and ethics board approval were obtained.

**Fig. 1:**
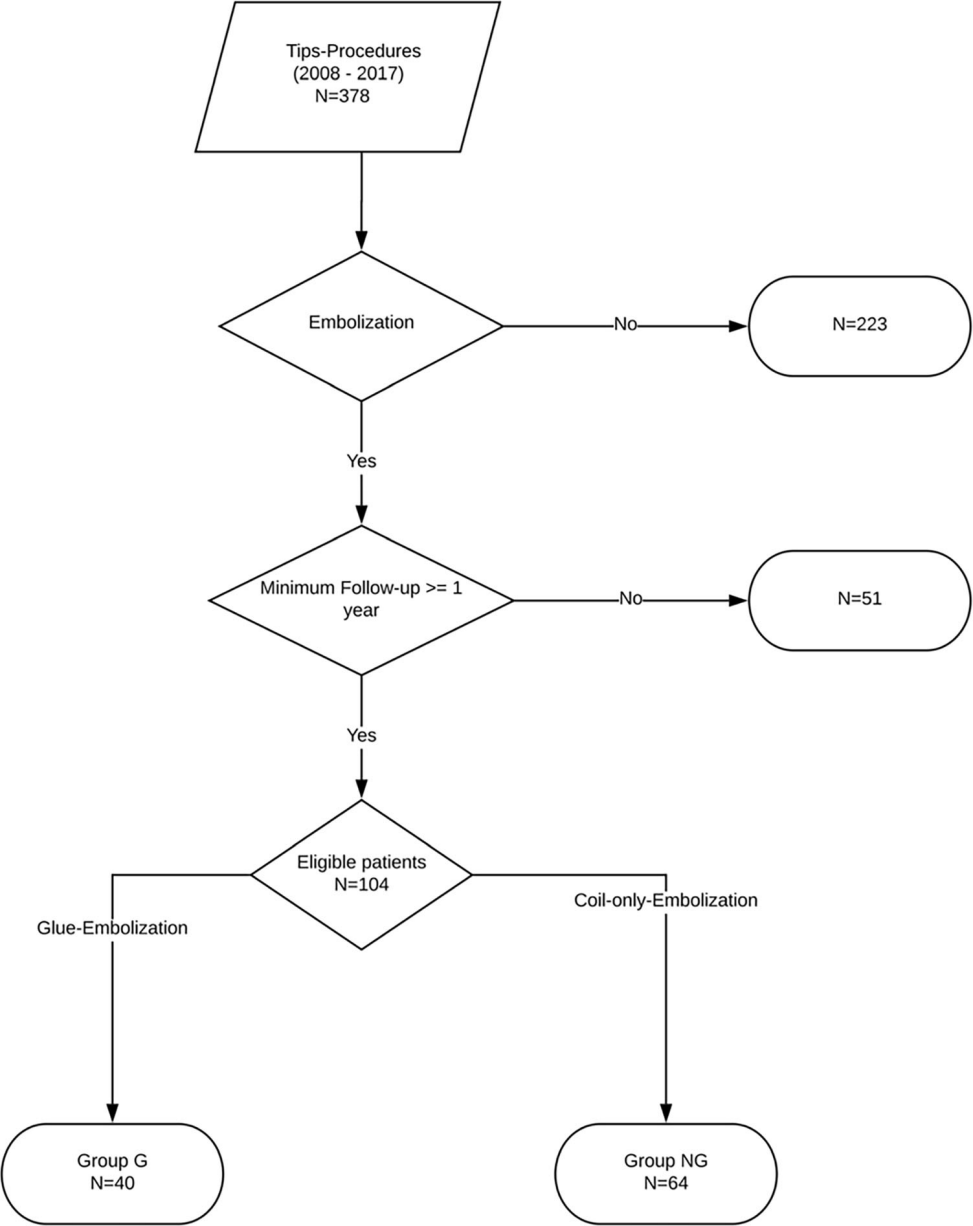
Patient inclusion—TIPS procedures with PTFE-covered stent

The detailed inclusion criteria were (Table 2): age above 18 years, decompensated liver cirrhosis, clinically significant portal hypertension (CSPH) (hepato-portal venous gradient (HPVG) > 10 mmHg), successful TIPS establishment with PTFE-covered stent endoprosthesis, embolization of gastroesophageal varices and a minimum follow-up period of one year (patients who were lost to follow-up during one year after embolization were excluded from the study). Between 2008 and July 2017, 378 patients received a primary TIPS procedure with PTFE-covered endoprosthesis at our facility. In total, 155 patients received concomitant embolization during TIPS. In total, 51 patients were excluded due to follow-up period of less than one year. In total, 104 patients (67 males; 37 females; *p* = 0.69) met our inclusion criteria (Fig. 1). Acute-on-chronic liver failure (ACLF) was defined as suggested by European Association for the Study of the Liver (EASL) guideline [5, 22], which represents laboratory parameters of liver, kidney and coagulation systems as well as cardiopulmonary parameters and hepatic encephalopathy.

**Table 2:** Inclusion and exclusion criteria for TIPSS placement and GEV embolization (HPVG = hepatic portal venous gradient

	Inclusion	Exclusion
TIPS placement	Recurrent GEV bleeding	HPVG < 10 mmHg
	Refractory ascites	Severe hepatic encephalopathy
		Bilirubin blood concentration > 2 mg/dl
GEV embolization	Persistent GEV filling on angiogram after TIPSS establishment	No filling of GEV after TIPSS in patients

Primary outcome parameters were six-week and one-year survival. Six-week overall survival was assessed as a surrogate for treatment failure as proposed by the international Baveno working group [23]. Secondary outcome parameters were treatment failure, ACLF development, variceal rebleeding and hepatic encephalopathy (as per West Haven criteria). Patients were divided into two groups: glue embolization ± coils (group G, glue) and coil-only embolization (group NG, no glue).

### Statistics

Since MELD, age and indication for TIPS are known factors that influence the outcome and were not evenly distributed between the groups, Cox regression analysis has been adjusted for these parameters.

Survival analysis was performed using Kaplan–Meier curves with log-rank tests as well as adjusted Cox regression analysis, group comparisons by t tests for independent samples (for patient age), Fishers exact test for TIPS indication as well as Mann–Whitney U test for all other single parameters. Statistical analysis and data plotting were performed using SPSS 27 (IBM, New York, USA).

### TIPS and embolization procedure

The procedures were performed by two interventionists (DT, CM)—both with more than 10 years of experience in interventional radiology. Before TIPS a contrast-enhanced CT scan was performed for procedure planning in order to discern the vascular anatomy (splenorenal shunt; portal vein thrombosis, etc.). TIPS establishment was performed using an ultrasound and fluoroscope-guided technique. After successful transjugular puncture of the portal vein, a 5 French marked pigtail catheter (Cordis medical; Bloomington IN, USA) was introduced into the portal vein (PV) and advanced to the confluence (CO) of superior mesenteric vein (SMV) and splenic vein (SV) to perform initial pressure measurement and contrast angiography (at CO and in SV) to assess gastroesophageal varices and length of stent to be placed. Shunt establishment was performed by deployment of a PTFE-covered stent (Gore Viatorr endoprosthesis, W.L. Gore Medical, Flagstaff, Arizona, USA). Dilatation of the stent was done according to the portosystemic pressure gradient (PSPG) to achieve an adequate decrease in PSPG with a target of 8–12 mmHg. After TIPS creation, an 5F pigtail catheter is placed into the SV and a standardized contrast angiography (flow: 15 ml/s; contrast volume: 30 ml) is performed. If varices were still present, embolization of these was performed using 3–14-mm (0.035 in) pushable coils (Cook medical), glue (mixture (1:3) of n-butyl-cyanoacrylate (Histoacryl, B. Braun AG, Melsungen, Germany) and lipiodol (Guerbet medical; Paris; France), or both at the discretion of the interventionist. A 4F catheter was placed into the varices and either glue was injected, or coils have been pushed through the catheter. In combined embolization first some coils have been inserted into the varices in order to reduce flow and prevent the then applied glue from “floating away” into the azygos vein and lungs. Embolization was performed until the varicose veins could not be detected on contrast angiography performed at the same site and equal standardized injection parameters.

## Results

### Patient cohort

Patient characteristics are shown in Table 1. Mean age was 55.93 years (SD 12.51). Patients were predominantly male (64%) with a mean MELD of 12.59 (SD 5.39). Child–Pugh stage did not show sig. differences between groups (*P* = 0.71). Indications for TIPS establishment were refractory ascites (*n* = 33) and variceal bleeding (*n* = 71). CT scans prior to embolization discerned 7 splenorenal shunts (*n* = 4 NG; *n* = 3 G; *p* = 0.55). In 76 patients endoscopic evaluation prior to TIPS was performed (Table 1) without significant differences between both groups (*P* = 0.81).

Embolization was performed using glue (*n* = 40) (group G) or coils-only (*n* = 64) (group NG, no glue). In 18 patients coils and n-butyl-cyanoacrylate were employed. There was no observed difference in PSPG (NG: 8 mmHg; G: 10 mmHg; *p* = 0.12) and portal venous pressure (NG: 19 mmHg; G: 21 mmHg; *p* = 0.49) after TIPSS. On average 8.34 coils have been used in group NG and 2.9 coils in group G (*p* =  < 0.0001). No difference in mean fluoroscopy time could be observed (NG = 35.28 min; G = 34.36 min; *p* = 0.87).

### Embolization and mortality

Results of univariate and multivariate analysis are shown in Table 3. Univariate analysis showed significant influence on overall survival for age (*p* = 0.01), MELD at TIPS (*p* = 0.001) as well as the MELD score subparameters (serum bilirubin (*p* = 0.001) and creatinin (*p* = 0.002) and INR (*p* = 0.017)) as significantly associated with overall survival. The indication for TIPS (i.e., refractory ascites and recurrent GEV bleeding) did not show any correlation (*p* = 0.998) but has been described as influential in prior studies [6].

**Table 3:** Results of multivariate and univariate analysis (PAR = patients at risk; HR = hazard ratio; PSP = portosystemic pressure gradient; PVP = portal venous pressure)

	Univariate				Multivariate			
	HR	95% CI		Sig	HR	95% CI		Sig
*Overall survival (1 year) (PAR = 27)*								
*Group*								
NG	2.483	1.002	6.155	0.042	3.333	1.193	9.312	0.022
G	Reference				Reference			
Patient age (years)	1.050	1.012	1.090	0.009	1.052	1.009	1.096	0.017
MELD score	1.108	1.054	1.166	< 0.001	1.144	1.072	1.220	< 0.001
PSP gradient after TIPS (mmHg)	0.980	0.907	1.060	0.622				
PSP gradient before TIPS (mmHg)	0.980	0.907	1.060	0.259				
PVP after TIPS (mmHg)	1.015	0.960	1.075	0.595				
PVP before TIPS (mmHg)	0.974	0.922	1.030	0.361				
Variceal bleeding before TIPS								
No	1.001	0.449	2.228	0.998	0.757	0.330	1.740	0.513
Yes	Reference				Reference			
*Overall survival (6 weeks)(PAR = 16)*								
*Group*								
NG	2.898	0.826	1.017	0.097	6.945	1.492	32.333	0.014
GG	Reference				Reference			
Patient age (years)	1.032	0.986	1.081	0.17	1.026	0.974	1.080	0.337
MELD score	1.157	1.085	1.234	< 0.001	1.224	1.112	1.347	0.000
PSP gradient after TIPS (mmHg)	0.986	0.890	1.092	0.791				
PSP gradient before TIPS (mmHg)	0.963	0.886	1.048	0.383				
PVP after TIPS (mmHg)	1.059	0.986	1.138	0.114				
PVP before TIPS (mmHg)	1.019	0.947	1.096	0.618				
Variceal bleeding before TIPS								
No	0.447	0.127	1.570	0.209	0.383	0.105	1.403	0.148
Yes	Reference				Reference			
ACLF (6 months)(PAR = 20)								
Group								
NG	1.576	0.606	4.103	0.351	3.243	1.062	9.900	0.039
G	Reference				Reference			
Patient age (years)	1.020	0.983	1.059	0.291	1.017	0.971	1.065	0.477
MELD score	1.180	1.110	1.253	< 0.001	1.228	1.135	1.328	0.000
PSP gradient after TIPS (mmHg)	0.969	0.882	1.064	0.504				
PSP gradient before TIPS (mmHg)	0.944	0.877	1.016	0.125				
PVP after TIPS (mmHg)	1.033	0.970	1.100	0.315				
PVP before TIPS (mmHg)	0.997	0.936	1.061	0.914				
*Variceal bleeding before TIPS*								
No	0.653	0.237	1.797	0.409	0.509	0.178	1.455	0.208
Yes	Reference				Reference			

**Fig. 2:**
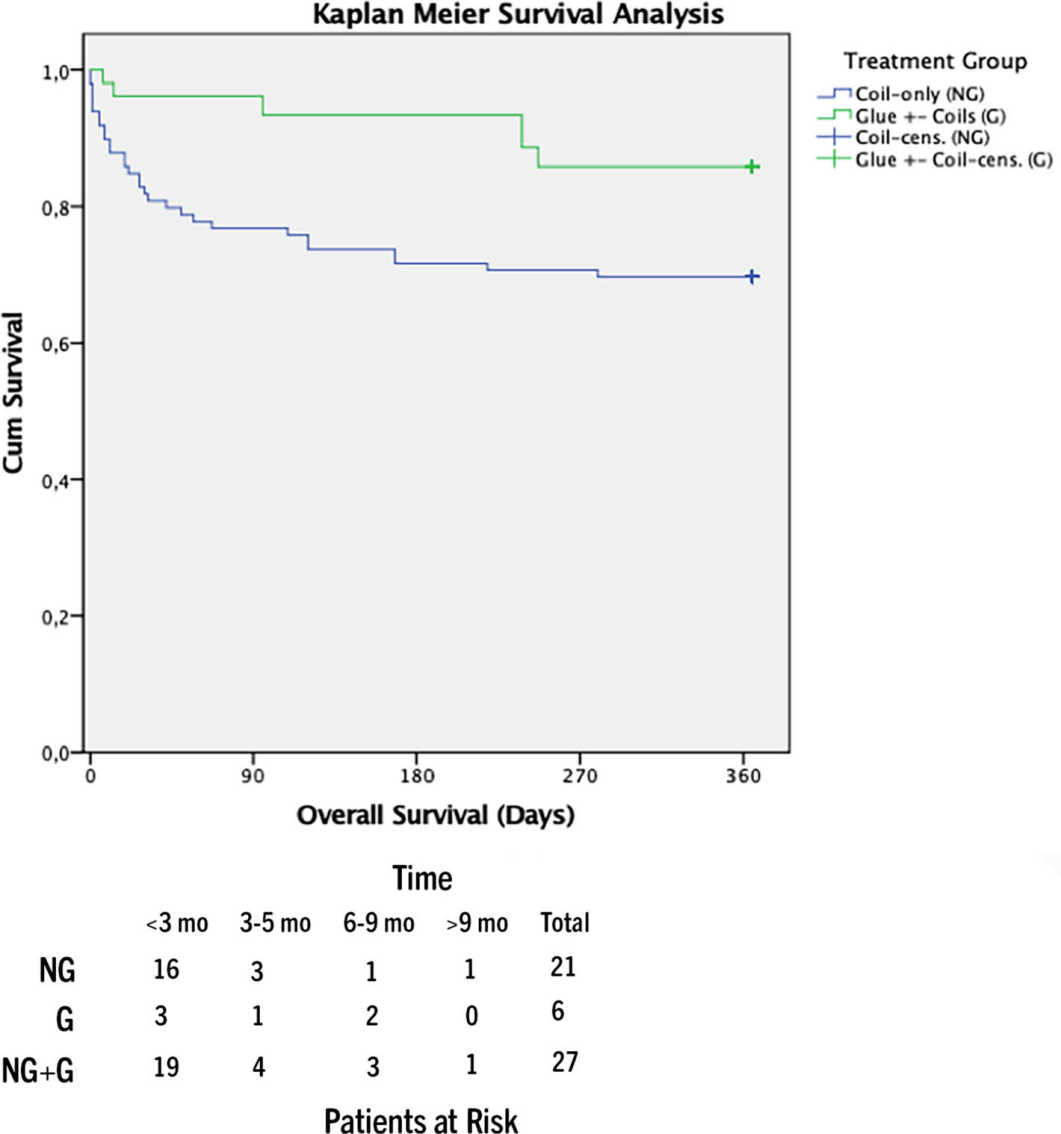
One-year mortality analysis glue vs coil-only embolization (p = 0.022; HR = 3.33) (mo = months)

**Fig. 3:**
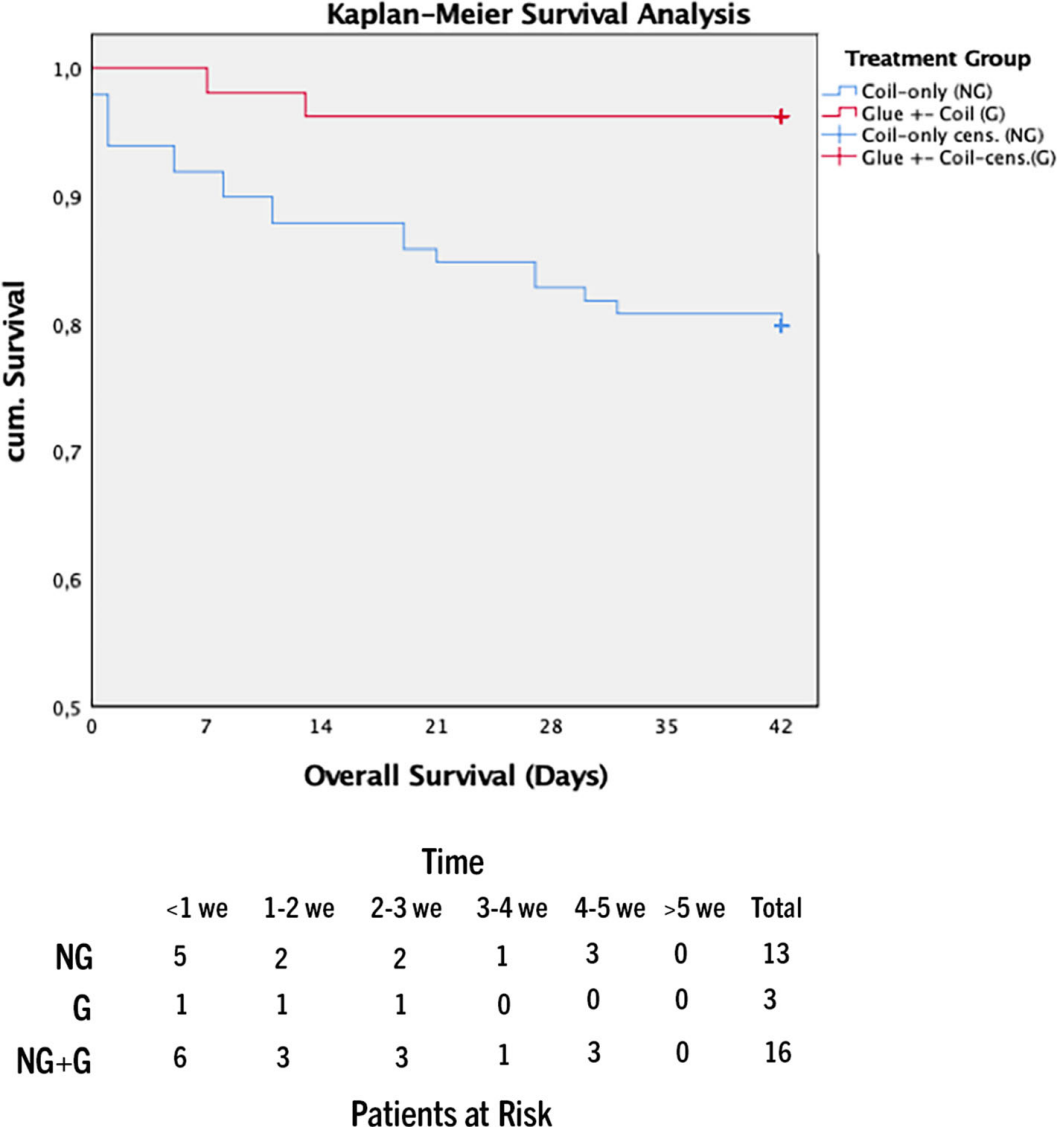
Six-week mortality analysis glue vs coil-only embolization (p = 0.014; HR = 6.95) (we = weeks)

**Fig. 4:**
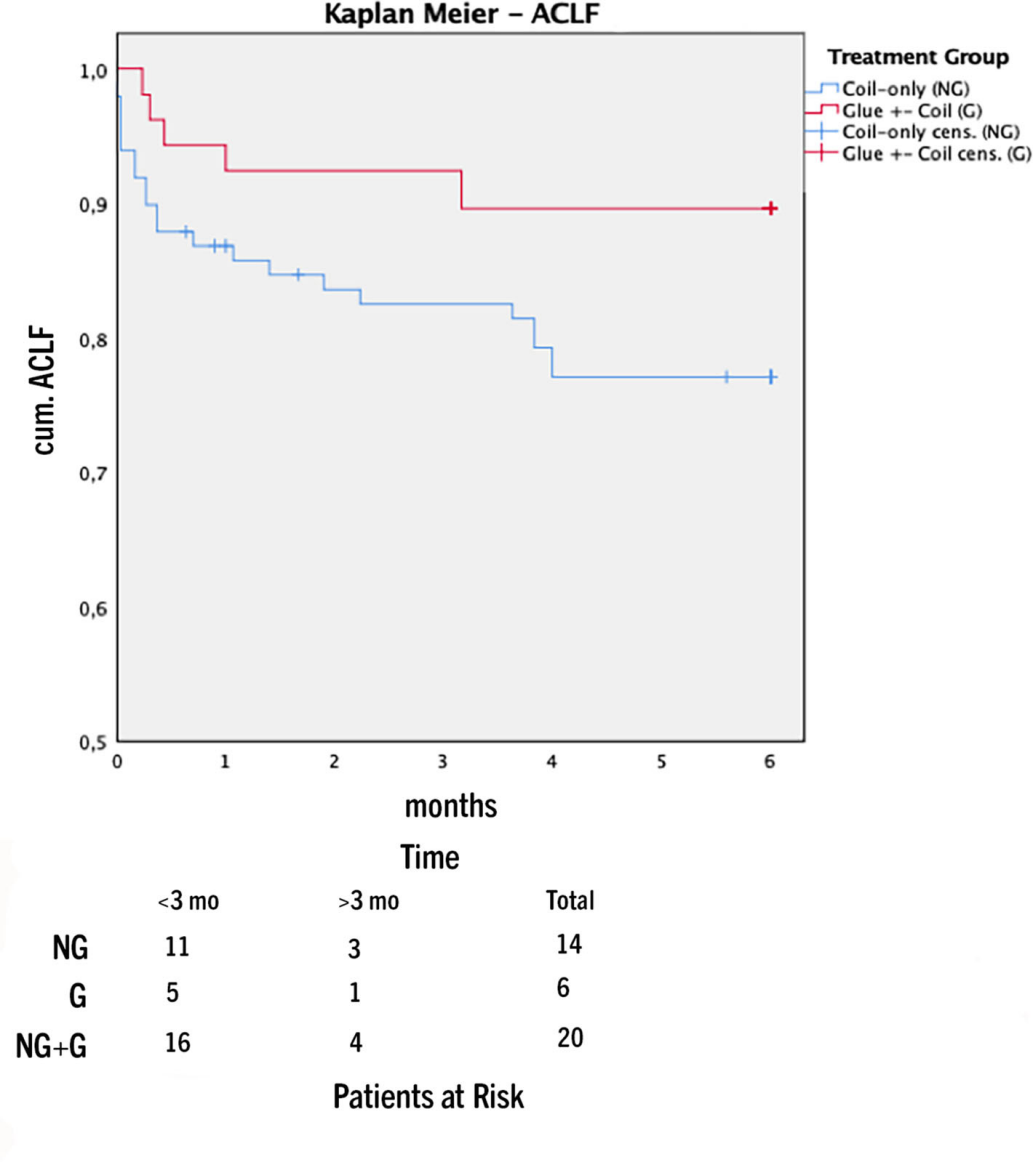
Acute-on-chronic liver failure (CLIF-ACLF)—glue vs coil-only embolization (p = 0.039; HR = 3.24) (mo = months)

To account for these potential confounders Cox regression adjustment for TIPS indication (refractory ascites/variceal bleeding), age and MELD score was performed. Causes of death are shown in Table 1. Patients who received coil-only embolization (group NG) had significantly higher (21 deaths) one-year mortality compared to group G (6 deaths) (p = 0.022; HR = 3.33) (Fig. 2). Six-week survival as a surrogate of treatment failure was significantly lower in group NG (*n* = 13) compared to group G (*n* = 3) (*p* = 0.014; HR = 6.95) (Fig. 3). Development of ACLF was significantly higher in group NG after 6 months (NG: *n* = 14; G: *n* = 6) (*p* = 0.039; HR = 3.24) (Fig. 4).

A subgroup Kaplan–Meier analysis of subgroups with variceal bleeding (*n* = 71) and refractory ascites (*n* = 33) as TIPS indication showed 18 deaths in the variceal bleeding subgroup (NG: *n* = 13 (est. surv: 264d) vs. G: *n* = 5 (est. surv: 317d); *p* = 0.135) within one year and 13 deaths within six weeks after TIPS (NG: *n* = 10 (est. surv: 34d) vs. G: *n* = 3 (est. surv: 39d); *p* = 0.116). ACLF during the first six months showed no significant difference (*n* = 15; NG = 9 (est. ACLF free time = 4.9 mo) vs. G = 6 (est. ACLF free time = 5 mo); *p* = 0.733).

In the sub-cohort with refractory ascites as TIPS indication group G showed a trend for longer 1-year survival compared to group NG (NG: *n* = 8 (est. surv: 267d) vs. G: *n* = 1 (est. surv: 352d); *p* = 0.142) and at six months (NG: *n* = 3 vs. G: *n* = 0; *p* = 0.243) in the glue group could also be observed. Also, development of ACLF during 6 months was non-significantly more pronounced in the NG subgroup (NG = 5; G = 0; *p* = 0.107).

However, in all of these sub-evaluations no significant difference could be seen, probably due to the reduced patient number in each sub-cohort.

### Embolization and complications

Occurrence of episodes of HE as defined by West Haven criteria in group G and group NG was not significantly different (15 (37%) vs. 22 (34%); *p* = 0.75) during one-year follow-up. Rebleeding rates in both groups (G: *n* = 3; NG: *n* = 6; *p* = 0.74) were very low as expected. However, rebleeding was fatal in some cases (G: *n* = 2; NG = 3; *p* = 0.94).

Post-TIPS CT scans were not routinely performed. However, on performed post-TIPS CT scans small glue material particles were found inside the lungs in group G. None of the cases had clinical signs of pulmonary embolism (CIRSE grade 1).

One severe non-target embolization in group G was observed. Glue material caused paradox embolism into the brain via a patent foramen ovale and caused an non-fatal stroke. The patient died from septic complications during the follow-up period (CIRSE grade 5).

No adverse events have been reported in group NG.

## Discussion

The present study evaluated the concomitant use of fluid embolization of GEV during TIPS compared to coil-only embolization.

It is an established fact that closure of GEV in patients with variceal bleeding in combination with TIPS lowers rebleeding rates [13, 24]. Moreover, recent data suggest presence of SPSS impacts outcome of cirrhotic patients [15], suggesting that efficient, overall shunt reduction is warranted in this patient group. However, different embolization materials (fluid and non-fluid) may show differences regarding their overall effectiveness [10].

Our data suggest that embolization of gastroesophageal varices with fluid embolization material (i.e., a mixture of n-butyl-cyanoacrylate and lipiodol) during TIPS establishment may reduce mortality, treatment failure and may also slow deterioration of liver function compared to coil-only embolization.

**Fig. 5:**
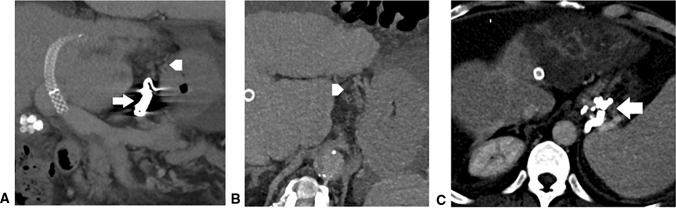
a–c: (a) CT after coil embolization of coronal vein (arrow) with distal not closed varix vessels (arrowhead) (cor. MPR); (b) distally not closed varix vessels (arrowhead) (ax); (c) CT after glue embolization of varix vessels forming “cast-like” formation (arrow)

**Fig. 6:**
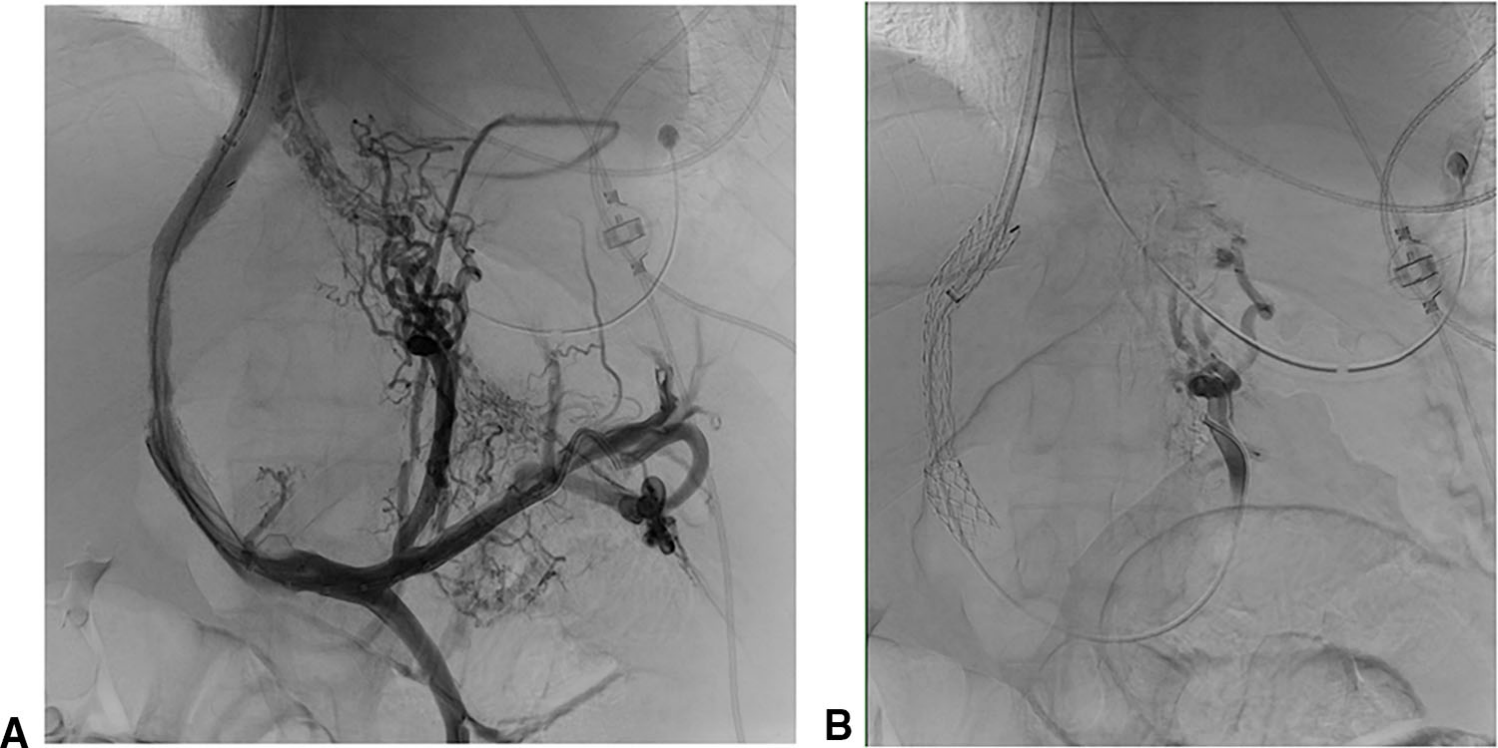
a + b: Contrast angiography showing large variceal network prior (a) and after glue embolization (b)

One explanation for this finding may be due to the physical characteristics of fluid embolization materials which disseminate into the network of GEV collaterals more easily and thoroughly—thus, leading to a cast-like formation accumulating in the periphery of GEV. In contrast, coil embolization facilitates a local closure only in major inflow vessels, where coils are deployed, but not the entire variceal network (Figs. 5, 6). Therefore, this embolization method might be more prone to incomplete occlusion of the inflow into EVs and refilling of the GEV from other collateral portal feeder vessels. This is supported by previously published work by Lakhoo et al. [17] in which the authors describe that in patients who underwent embolization of GEV without usage of glue embolization (i.e., coil and plug embolization), 65% of the varices remained patent, which was likely due to recruitment of other afferent supplying vessels to the variceal network after successful closure of the primary inflow vessel.

Therefore, persisting portosystemic shunting with coil-only embolization might be a feasible pathophysiological explanation for our results, which are further substantiated by previous studies which did show that patients with TIPS and patent portosystemic collaterals exhibited worse rebleeding and HE rates [15, 24, 25].

Surprisingly, although according to our data the examined groups differed in one-year survival, a significant difference in rebleeding rate could not be observed. This is in concordance with previous published data showing that reduction of rebleeding is only one of many factors TIPS influences mortality in the follow-up period [6].

It has been shown that TIPS increases effective blood volume and therefore improves systemic hemodynamics and increases renal perfusion [26]. Blood contained in GEV is also lost to effective systemic vascular flow; thus, reduction of GEV volume accomplished by cast-like glue embolization filling may further contribute to systemic hemodynamics as compared to coil-only embolization.

In our cohort there was no significant difference in rates of HE episodes between both embolization methods. In fact, recently it has been shown that HE rates were not different between closed and not embolized/patent varices in TIPS patients, which is supported by our results [24]. Moreover, a recent study suggested that higher HE rates are only found in patients after TIPS who had large (> 8 mm diameter) patent spontaneous portosystemic shunts (SPSS). In patients whose SPSS have been small, no significant worsening of HE-specific outcome could be seen [14]. It is therefore conceivable that regarding HE rates full cessation of flow inside GEV is not necessary and embolization of major GEV-feeding vessels is able to sufficiently reduce flow to reduce HE rates regardless of material employed.

On the other hand, usage of fluid embolization materials especially glue is more challenging in application. Also, these materials are more prone to major complication and non-target embolization. Coils are more precise and easier to apply especially if detachable coils are used. In summation employment of glue needs more experience and specialized training. Also prices of glue and coils (pushable and detachable) in different countries may vary significantly so that decision for either embolization method needs to take these regional conditions into account.

Our study has several limitations. Firstly, even though these patients were from the prospective NEPTUN cohort, this is a retrospective analysis. We examined a heterogeneous group of patients with different indications for TIPS (refractory ascites and variceal bleeding), which carry a different overall prognosis. It is known that these indication groups feature a different overall prognosis [6]. Furthermore, the NG group was older compared to the G group. However, we addressed these confounders in this work by adjusting the Cox regression analysis. Although our results are convincingly significant, further studies consisting in a more homogenous population would be desirable. Randomized prospective matched cohorts are desirable and should be done in further studies but are difficult to obtain in an interventional emergency setting.

Additionally, embolization method selection was performed at the operator’s discretion. This was mainly in regard to material availability of lipiodol and correctly sized coils as well as proficiency of material preparation by the radiology assistant personnel. This may introduce a slight selection bias. However, since these influencing factors have been independent of the patient, it is therefore conceivable that the impact on procedure outcome should be evenly distributed between the groups.

Although we took great efforts in standardizing our embolization approach regarding which GEV to embolize and when embolization is finished (standardized catheter positions and contrast injection parameters), the nature of portal hemodynamics introduces a slight fuzziness in these definitions. Since this is independent of embolization method, it is valid to assume that there should be no influence on outcome parameter evaluation comparing glue usage.

Also, some data which could be influential on outcome could not be validly assessed in all cases. Endoscopic grading of GEV prior to TIPS was not possible in 28 cases, and distinct quantitative parameters of GEV (diameter, etc.) have not been assessed. Since not sig. difference between groups in GEV grading could be observed, it is assumable that the remaining cases would show an equivalent distribution.

Another limitation has been that some data which may have given us more understanding of embolization effectiveness like volume of glue injected, DAP, success of ascites control have not been thoroughly validly documented. Therefore, some opportunities of gaining insight have been missed and should be captured in further prospective studies.

Although clinical apparent and initial complications were documented, occult non-significant adverse events, like small lung emboly, have been registered only incidentally on random post-TIPS CT scans and not logged quantitatively. These should be done in more standardized fashion in further investigations.

## Conclusion

In conclusion this study shows for the first time that closure of gastroesophageal varices in patients undergoing TIPS utilizing glue embolization material may improve overall survival and may be preferable over coil embolization alone.
